# Adapting and Validating the Global Physical Activity Questionnaire (GPAQ) for Trivandrum, India, 2013 

**DOI:** 10.5888/pcd13.150528

**Published:** 2016-04-21

**Authors:** Elezebeth Mathews, Deborah Salvo, Prabhakaran Sankara Sarma, Kavumpurathu Raman Thankappan, Michael Pratt

**Affiliations:** Author Affiliations: Deborah Salvo, Michael and Susan Dell Center for Healthy Living, The University of Texas Health Science Center at Houston, School of Public Health–Austin Regional Campus, Austin, Texas; Prabhakaran Sankara Sarma, Kavumpurathu Raman Thankappan, Achutha Menon Centre for Health Science Studies, Sree Chitra Tirunal Institute for Medical Sciences and Technology, Trivandrum, Kerala, India; Michael Pratt, Hubert Department of Global Health, Rollins School of Public Health, Emory University, Atlanta, Georgia.

## Abstract

**Introduction:**

A limitation of the Global Physical Activity Questionnaire (GPAQ) in assessing physical activity in India is that it does not capture the diversity of activities across cultures and by sex. The purpose of this study was to culturally adapt and validate the GPAQ by using an accelerometer in Thiruvananthapuram City, India.

**Methods:**

We developed a modified version of the GPAQ by adding a physical activity chart specific to the locale. We identified local physical activities through in-depth interviews, group discussions, and observation, and used Actigraph GT3X accelerometers to validate the modified GPAQ for a subsample of 47 women. Participants were drawn from a cross-sectional survey of 1,303 women aged 18 to 64 years, selected by multistage cluster sampling. Spearman rank correlation coefficients and intraclass correlation coefficients (ICC) were calculated to determine the correlation and level of agreement in moderate-to-vigorous physical activity (MVPA) on the basis of accelerometer measurement and the modified GPAQ.

**Results:**

The correlation for MVPA between the modified GPAQ (overall) and the accelerometer (non-bouted MVPA) was 0.69 (95% confidence interval [CI], 0.39–0.85) with a moderately high ICC of 0.78 (95% CI, 0.56–0.90). The correlation for MVPA between the modified GPAQ and the accelerometer-based MVPA within bouts of at least 10 minutes was 0.60 (95% CI, 0.26–0.80) with an ICC of 0.55 (95% CI, 0.20–0.77) indicating a moderate level of agreement.

**Conclusion:**

The GPAQ can be used for assessing physical activity among women in India, and its adaptation and validation may be useful in other low-income or middle-income countries where activities are diverse in type and intensity.

## Introduction

Assessing physical activity for epidemiological studies is challenging. Although self-report is widely used because of low cost, ease of implementation, and low participant burden, it has important limitations for estimating physical activity accurately (1). Self-report tends to overestimate or underestimate actual physical activity, energy expenditure, and inactivity levels because of issues of recall and response bias (eg, social desirability, inaccurate memory) and the inability to capture data on the absolute level of physical activity (2).

In some large population-based studies, pedometers and accelerometers are used as objective measures of physical activity. Pedometers measure steps taken, whereas accelerometers record movement as “acceleration counts,” estimating the intensity and duration of bouts of activity and energy expenditure. Pedometers are more feasible for use in large observational and intervention studies in low-to-middle income countries because of low cost and immediate feedback to the participant; however, pedometers have an error rate of up to 30% or more (3) in estimating energy expenditure compared with 2.5% for accelerometers (4). Hence, accelerometers are considered a standard criterion validity tool for objective physical activity assessment.

Several questionnaires were developed worldwide, most in high-income countries and a few in low-income and middle-income countries such as India (5, 6). Use of different questionnaires for assessment of physical activity in different settings makes the findings inconsistent and poorly comparable. To minimize the intercountry and within-country differences in physical activity assessment, the World Health Organization (WHO) developed a Global Physical Activity Questionnaire (GPAQ) (7). The GPAQ assesses physical activity in multiple domains (work, travel and leisure) and is used widely worldwide.

The GPAQ’s validity and reliability were initially estimated by using pedometers and accelerometers as part of a 9-country study that included India (8). Pedometer validation of the GPAQ in India (8) showed a moderate correlation coefficient (Spearman ρ = 0.35) for total physical activity (ie, total minutes of physical activity) and total pedometer counts per day. GPAQ pedometer-validation in other countries, such as China, Ethiopia, Indonesia, and Japan, also showed significant but weak to moderate correlations, with a Spearman ρ ranging from 0.23 to 0.35 for total physical activity time. Accelerometer-based validation of the GPAQ (8) was undertaken in China and South Africa and showed significant but weak to moderate correlation coefficients (0.23–0.40) for sedentary and moderate to vigorous physical activity (MVPA) in China and a significant correlation (0.26) for vigorous-intensity activity in South Africa. Level of agreement between the findings of GPAQ and accelerometer- or pedometer-based assessment was not ascertained.

Further validation studies with accelerometers, beyond the initial 9-country study, were conducted with adults in Malaysia (9), South America (10) and Europe (11). Validation with accelerometers in Malaysia (9), the United States (10, 12) and European countries (11) showed weak to moderate correlation for MVPA among adults (0.26– 0.48).

WHO incorporated the GPAQ into the WHO STEP wise approach to surveillance (STEPS) of noncommunicable disease risk factors (13) and recommended the use of “show cards” to illustrate different kinds of moderate-intensity and vigorous-intensity physical activity. Respondents identify activities illustrated in the show card based on the intensity of the activity and report the cumulative duration of activities per week. Studies from India (14,15) and other parts of the world (16) indicate that people tend to perceive low-intensity activity as moderate, and moderate-intensity activity as vigorous, thereby over-reporting their intensity of physical activity. Furthermore, intensities of the same activity were perceived differently by different people (14). The prevalence of meeting the WHO’s recommendations for physical activity of 150 minutes per week of MVPA, estimated using the GPAQ in India, ranged from 7.3% to 93.2% (17–21). The wide within-country variation of meeting WHO’s recommendations could be due to poor validity of the instrument for these populations or due to lack of sensitivity to the diverse sociocultural and sex differences of the people who engage in the activities.

The GPAQ has several advantages because of its focus on generic domains of activities such as work, transportation, and leisure, which enhance its applicability to multiple settings (8). Furthermore the GPAQ is sufficiently concise for physical activity surveillance and is standardized internationally, enabling within-country and between-country comparisons (8). Although the GPAQ has high credibility and is widely used for international comparisons of physical activity, one major limitation for assessments in India is that it does not capture the diversity of activities across cultures and by sex (6). To make a more precise physical activity measurement for South India, we adapted the GPAQ to Indian culture and validated it with the accelerometer criterion measurement of physical activity assessment. We conducted our study in Thiruvananthapuram City in South India. Because our study was part of an ongoing intervention trial targeting women, the modified GPAQ was developed and validated for adult women only.

## Methods

### Sample frame

Participants were a subsample (N = 47) of 1,303 women aged 18 to 64 years who participated in a published cross-sectional survey to estimate the prevalence of physical activity (22). Participant profiles of 1,303 women were as follows: 327 (25.1%) were younger than 25 years, 649 (49.8%) were 25 to 54 years, and 327 (25.2%) were 55 years or older; 657 (50.4%) had some secondary education or less, and 646 (49.6%) had completed secondary education or more; 277 (21.3%) were employed, and the remaining 78.7% were unemployed. The mean activity duration (minutes per week) for the 1,303 the women was 369.31 (standard deviation [SD], 350.9) of moderate-intensity activity, and 12.54 (SD, 35.9) of vigorous-intensity activity. The modified GPAQ was administered to all 1,303 women. However, the validation with accelerometer was done only on data of the 47 women whom we selected by purposive sampling. We selected women who were willing to participate in the accelerometer-validation procedure for the study.

Accelerometer use for health research was new in India when this study was conceptualized. Therefore, we conducted this as a feasibility study. The validation of the modified GPAQ with accelerometers was intended for a subsample of up to 50 participants, which was reported as adequate for a reliability study (23)

### Development and validation of the Modified Global Physical Activity Questionnaire

The GPAQ was culturally adapted to be effective in assessing the amount of physical activity for South Indian women. Our study consisted of 2 stages.

#### Stage 1: Develop the modified version of the Global Physical Activity Questionnaire

Misperception about the intensity of various physical activities presents a challenge to studies in low-income and middle-income countries such as India given the diverse range of physical activities. This challenge could be overcome by measuring the frequency and duration of each activity performed locally at work, for travel, and during leisure time thereby improving the performance of GPAQ. The modified GPAQ (Appendix) was developed by supplementing the original GPAQ with an activity chart. The content of the core questions in the original GPAQ was not changed but was supplemented with an activity chart that provided contextually appropriate examples of activities of different domains and intensity levels. The activity chart is embedded in the modified GPAQ under moderate and vigorous intensities at work and for travel. The activity chart was made in 2 phases. Initially we identified locally specific activities at work, for travel, and during leisure through interviews of women in their community, through group discussions (15), and by observation of women’s everyday activities.

 Seven in-depth interviews, 4 focus group discussions, and 2 observations were made among women from the sample frame. Among women who participated in interviews, 4 were aged 24 to 45 years and 3 were aged 46 to 60. Five women had completed secondary education or less, 3 had completed education beyond secondary level, and 2 were employed (ie, worked outside the home). Most household activities included in the activity chart were of moderate intensity, with a few vigorous-intensity activities reported in unorganized work-place settings by the 2 employed women. Focus group discussions published elsewhere (15) explored activities commonly performed by women in general, including barriers to and facilitators of those activities. Two unstructured day-time observations were made on weekends for an average duration of 3 hours each to get insights into the nature and duration of the physical activities women engaged in. The findings from all qualitative techniques used were corroborated with input from experts in the fields of physical activity and community-based research.

Activity intensity was later classified as light, moderate, or vigorous on the basis of the compendium of physical activity (24). Activities at work and for travel were incorporated into the modified GPAQ as examples of type and intensity. The section on recreational activities remained the same as that of the original GPAQ because no leisure activities of moderate or vigorous intensity, apart from walking, were elicited from interviews and discussions. For example, the GPAQ asks responders whether they engaged in work that involves vigorous-intensity activity and that causes large increases in breathing or heart rate for at least 10 minutes continuously. If the response is “yes,” the participant is asked about the duration (minutes or hours) and frequency (number of times per week) of all the locally specific activities of vigorous intensity done by women. Examples from the modified GPAQ include carrying, loading, or stacking wood; chopping wood or splitting logs; drawing water from the well; and carrying heavy loads such as bricks.

For locally specific work activities of moderate intensity lasting more than 10 minutes, participants were probed on the duration (minutes or hours) and frequency (number of times per week) of these activities. For travel from one place to another of more than 10 minutes’ duration, participants were probed on the duration (minutes or hours) and frequency (number of times per week) of walking or bicycling. The summary estimate of total physical activity was the sum of the products of frequency, duration, and intensity of MVPA in each domain. A standard value of 4 METs (metabolic equivalent of tasks) was assigned for moderate-intensity activities at work or leisure, and a value of 8 METs was assigned for work or leisure of vigorous intensity. Travel to and from places either by walking or bicycling was assigned an intensity value of 4 METs.

The modified GPAQ was initially developed in English and translated into the local language (Malayalam) and back-translated to English before pretesting. Pretesting was done with 10 women (5 housewives in the study community, 2 students in a university, and 3 women employed in a government sector) aged 18 to 60 years (mean age, 39 y; standard deviation [SD], 12.9). Pretesting showed that the modified GPAQ was feasible for implementation because it was self-explanatory, the list of activities was comprehensive, and the time taken to complete the questionnaire was about 15 minutes.

The modified GPAQ was administered by accredited social health activists of the local community who invited study participants to a local community facility. Women were briefed on the study’s purpose and procedures. Those who were willing to wear an accelerometer for 7 consecutive days and who gave consent were included in the study. Although we aimed to validate the modified GPAQ with 50 women, only 47 women from 4 residents’ associations were willing to participate. The modified GPAQ was administered verbally because 4 of the participants were illiterate. Total time required to administer the modified GPAQ ranged from 10 to 20 minutes per person, depending on the person’s activity profile.

#### Stage 2: Put field work procedures for accelerometer data collection into action

We used Actigraph GT3X accelerometers to validate the self-reported modified GPAQ for the subsample of 47 participants drawn from a cross-sectional survey. After the self-reported physical activity assessment using the modified GPAQ was complete, women wore the accelerometer on their right hip for 7 consecutive days during waking hours. The devices were initialized using Actilife version 5.10.0.0 software (Actigraph LLC), and delivered in person to participants. The devices were programmed to start collecting data at 4:00 am of the following day using 60-second epochs and a 30 Hz sampling rate. Women were oriented to data collection procedures and were shown how to fasten the accelerometer to the waist on the right side using a waist band provided with the device. Weight and height were measured during the first visit by using a standard protocol and equipment (13). To enhance protocol compliance, a local resource person reminded the women about wearing the accelerometer every day. The accelerometers were collected in person after 7 days of data recording. This study was approved by the institutional ethics committee of Sree Chitra Tirunal Institute for Medical Sciences and Technology, Trivandrum, India.

### Data analysis

Data from the modified GPAQ were scored using SPSS version 17 (IBM Corp). Overall minutes per week of MVPA were derived by adding the products of duration (minutes) and frequency (number of times per week) of each of the reported work-related, travel-related, and leisure-related physical activities.

Accelerometer wear-time was verified using Actilife 6.0 (Actigraph, LLC). The data were determined to be valid if the accelerometer was worn at least 4 days with sufficient valid days. A valid day was defined as at least 10 hours of wear time; nonwear-time was defined as 60 consecutive zeroes (1 hour) or more. Data were scored using Freedson’s cut points for adults (25). Minutes per week of overall and bouted moderate-intensity and vigorous-intensity physical activity and MVPA were derived. Bouted was defined as activities of moderate or vigorous intensity occurring within a sustained period of time lasting at least 10 minutes, for which at least 80% of the time corresponded to MVPA. Therefore, 20% of the total bout duration was allowed for break periods (a break period being of lower intensity than the moderate physical activity cut point), to account for real-life situations in which a bout of physical activity is still taking place in spite of a brief interruption or decrease of intensity. If a bout had a single break with a duration of more than 2 minutes, the bout was considered to be interrupted. This allowed a maximum of 2 minutes of break time for every 10-minute period. This definition of bouts has been used previously (26). Overall and bouted physical activity was scored using MatLab 7.7 (The Math Works Inc) as described previously (26).

Because the self-reported and accelerometer-based physical activities were not normally distributed, Spearman rank correlation coefficients were calculated to determine the correlation between GPAQ and accelerometer-derived MVPA. The correlation of MVPA between self-reported and accelerometer-based overall minutes per week for both bouted and nonbouted activities was calculated. Level of agreement between self-report and accelerometer-derived physical activity was assessed for both bouted and nonbouted activities by obtaining the intraclass correlation coefficient (ICC). The cut-off used for the measures of agreement between the modified GPAQ and accelerometer-based physical activity was 0 to 0.19, no relationship; 0.20 to 0.39, low relationship; 0.40 to 0.59, moderate relationship; 0.60 to 0.79, moderately high relationship; and 0.80 to 1.0 high relationship (27). We used SPSS version 17 (IBM Corp) to perform statistical data analysis.

## Results

The mean age of the 47 women in this study was 46.4 years (SD, 3.1). Although accelerometer data and self-reported data were captured for 47 women, only 24 women (51.1%) met the criterion of 4 valid days (at least 10 valid hours per day) of accelerometer wear time. This left us with an analytic sample of 24 women. We found no significant differences in the sociodemographic profile of the women 

with valid data and the women with nonvalid data except among age groups (Table 1). 

**Table 1:** Sociodemographic Characteristics of Women (N = 47) With Valid or Nonvalid Data Participating in Study of Modified Global Physical Activity Questionnaire, Trivandrum, India ,2013

Characteristic	Total, n (%)	Valid Data^a ^(n = 24)	Invalid Data^b ^(n = 23)	*P* Value^c^
**Age, y**				
18–35	11(23.4)	2	9	.03
36–55	26 (55.3)	15	11	
≥55	10 (21.3)	7	3	
**Occupational status**				
Employed	08 (17.0)	2	6	.14
Unemployed	39 (83.0)	22	17	
**Education**				
<High school degree	06 (12.8)	3	3	>.99
≥High school degree	41 (87.2)	21	20	
**Marital status**				
Married	47 (100.0)	24	23	—^d^
Unmarried	0	0	0	

^a^ Valid refers to participants who had at least 10 hours of data on 4 or more days per week.·

^b^ Invalid refers to participants who did not meet the criteria for analysis.

^c^ Significant at *P = *.05. *P* values calculated using χ^2^ test.

^d ^Not applicable.

The mean duration of overall accelerometer-based nonbouted MVPA was 116.9 (SD, 76.4) minutes per week. Of the 24 women with valid data, only 15 had at least one bout of accelerometer-based MVPA, and mean of minutes-per-week of MVPA within bouts of at least 10 minutes was 40.20(SD, 54.4). The mean duration of vigorous-intensity activity was 0.18 (SD, 0.73) minutes per week. Mean duration of GPAQ-based MVPA was 137.3 minutes per week with no vigorous-intensity activity reported. Table 2 presents the physical activity profile of women with valid and invalid data captured through self-reports and modified GPAQ. 

**Table 2:** Physical Activity Among Women (N = 47) Participating in Study of a Modified Global Physical Activity Questionnaire With Valid (n = 24) or Nonvalid (n = 23) Data, Trivandrum, India, 2013

Physical Activity	Minutes per Week, Mean (SD)			
	Valid^a^	Invalid^b^	Total	*P* Value^c^
Self-reported as moderate	136.45 (85.2)	171.7 (143.8)	153.72 (117.6)	.20
Self-reported as vigorous	0	0	0	—^d^
Accelerometer-measured as sedentary	3,637.9 (806.0)	3,540.0 (787.5)	3,595 (789.9)	.68
Accelerometer-measured as moderate	116.7 (76.4)	127.1 (107.8)	121.2 (90.1)	.11
Accelerometer-measured as vigorous	0.18(0.7)	5.3 (16.3)	2.4 (90.1)	.001
Average daily accelerometer-measured	907.57 (119.8)	881.67 (157.6)	896.4 (136.1)	.17
Number of valid days	6.0 (1.0)	1.3 (1.0)	3.7 (2.6)	.89

Abbreviation: SD, standard deviation

^a^ Valid refers to participants who had at least 10 hours of data on 4 or more days per week.

^b^ Invalid refers to participants whose data did not meet the criteria for analysis.

^c^ Significant at *P = *.05. *P* values calculated by independent sample *t* test.

^d ^Not applicable.

The correlation for MVPA between the modified GPAQ and nonbouted accelerometer-based assessment was 0.69 (95% confidence interval [CI], 0.39–0.85). The level of agreement for overall MVPA between self- reported physical activity and accelerometer-based measurement was moderately high with an ICC of 0.78 (95% CI, 0.56–0.90).

The correlation for MVPA between the modified GPAQ and accelerometer-based assessment within bouts of at least 10 minutes was 0.60 (95% CI, 0.26–0.80). The level of agreement between self-reported and bouted accelerometer-based MVPA was moderate with an ICC of 0.55 (95% CI, 0.20–0.77).

The mean difference between self -reported MVPA and accelerometer-based overall MVPA (nonbouted) was 20.4 (SD, 10.3) minutes per week, whereas for bouted MVPA the difference was 97.1 (SD, 32.3) minutes per week (Figure). 

**Figure:**
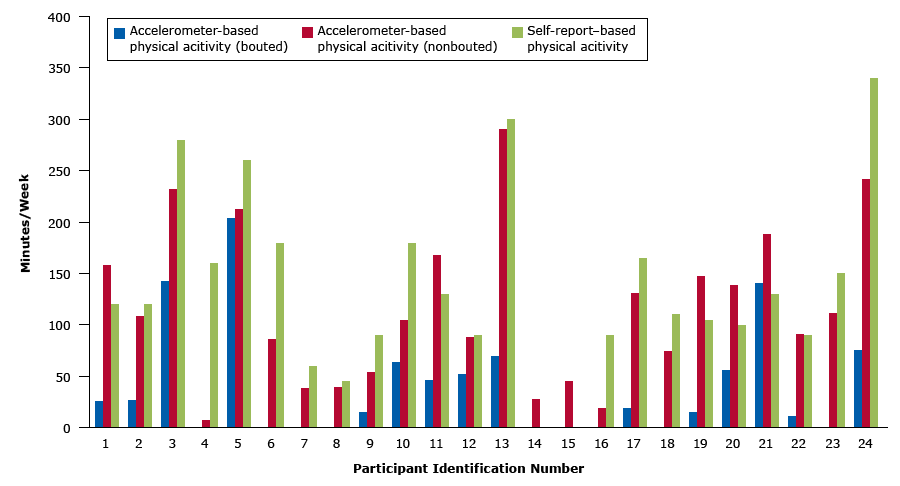
Comparison of minutes per week between moderate-to-vigorous physical activity measured by accelerometer and measured by self-report among 24 women with a minimum of 4 days (of at least 10 hours per day) of valid data. Accelerometer measurements are bouted and nonbouted. Bouted was defined as consisting of activities of moderate or vigorous intensity occurring within a sustained period of time and lasting at least 10 minutes for which at least 80% of the time corresponded to moderate-to-vigorous physical activity. Nonbouted is physical activity of shorter duration or lower intensity. A gap where a bar for physical activity might appear indicates zero minutes. Data for participants appear in no particular order. Abbreviation: MET, metabolic equivalent tasks. **Table:** 

Participant	Accelerometer-Based Physical Activity (Bouted), Minutes per Week	Accelerometer-Based Physical Activity (NonBouted), Minutes per Week	Self-Report–Based Physical Activity
1	25.7	158.7	120.0
2	26.8	108.5	120.0
3	142.3	232.2	280.0
4	0	7.0	160.0
5	204.2	212.3	260.0
6	0	86.3	180.0
7	0	39.0	60.0
8	0	39.6	45.0
9	15.0	54.0	90.0
10	64.2	105.0	180.0
11	46.2	168.0	130.0
12	52.0	88.0	90.0
13	70.0	290.5	300.0
14	0	28.0	0
15	0	45.5	0
16	0	19.0	90.0
17	19.3	131.3	165.0
18	0	74.7	110.0
19	15.4	147.0	105.0
20	56.0	138.8	100.0
21	141.0	188.0	130.0
22	11.0	91.0	90.0
23	0	112.0	150.0
24	76.0	242.0	340.0

The correlation for overall MVPA between the modified GPAQ and accelerometer measurements for women with at least 1 day of valid data was 0.56 (95% CI, 0.30–0.53; *P* = .001). The mean number of days was 4.12.

## Discussion

The validation of the self-reported modified GPAQ with accelerometer measurements showed that the modified version of GPAQ was sensitive to local cultural physical activity practices and was a good tool for assessing physical activity among women in Thiruvananthapuram City, India.

The mean duration in minutes per week of overall accelerometer-based MVPA in our study was lower (116.9 min; SD, 76.4) than that reported among women in another study of low-to-middle income women (175.2 min; SD, 7.5) (26).The mean duration in minutes per week of vigorous intensity activities (0.18) was negligible, and this finding is consistent with that of the other study (26). Women in our study engaged essentially in no physical activity of vigorous intensity, a consistent finding from both measurement instruments (self-report and accelerometers), contrary to the generalized perception of high levels of vigorous-intensity activities in low-to-middle income countries. This finding was corroborated by the findings from focus groups on the perceptions of physical activity among women in South India (15) in which women generally perceived that household activities were adequately active and reported not taking part in any leisure-time physical activity because it did not fit in gender and cultural norms.

Another important finding from this study was that self- reported physical activity was comparable with the overall minutes per week of MVPA measured by accelerometers, but not with MVPA within bouts of at least 10 minutes, although GPAQ asks participants to report only activity that lasts 10 minutes or more. This finding could be because, as reported by another study (24), people have more trouble recalling duration of physical activity than its intensity. The concept of bouts of physical activity and their importance for health benefits was not understood by study participants (15). Although further sensitizing Indian women to this concept might technically improve responses to GPAQ, it could actually reduce the concordance between minutes of overall physical activity between GPAQ and accelerometry measurements. The pattern of low levels of MVPA and negligible vigorous physical activity that we found among South Indian women could be a reason for the alarmingly high levels of overweight in this population (28) and the high prevalence of noncommunicable diseases (19,20).

Given that assessing physical activity by self-report and by accelerometry are conceptually different, it is not surprising that the two typically produce different estimates of MVPA. A systematic review of studies on validation of self-reported physical activity with accelerometer measurements showed that women were generally more likely than men to report higher levels of physical activity by self-report than were found through accelerometer measurements; the mean difference between the measurements was 138% (2). However, the over-reporting of 20.4 minutes per week of nonbouted MVPA and 97.1 minutes of bouted MVPA by self-report versus accelerometer measurement in this study was small compared with the findings of other studies (16). The small difference reported could be due to the inherent limitation of accelerometers for capturing data on upper-body movement or activities with limited movement of the center of body mass, such as washing clothes or washing dishes (2), which are common household chores among women in this study’s setting. Although the level of agreement between the modified GPAQ and the accelerometer-measured physical activity was moderate for the bouted physical activity and moderately high for nonbouted physical activity, agreement is higher than what has been observed in other GPAQ validation studies conducted worldwide (9–12). This finding suggests that the modified GPAQ is well adapted to capture the diversity in the household activities of our study population, and is sensitive to the culturally practiced physical activities among South Indian women.

This study has some limitations. The validation of self-reported physical activity with accelerometers was based on data from only 24 women of the original sample of 47. Most women wore the accelerometers for fewer than 3 days even after repeated reminders. Some women were dissuaded from wearing the accelerometers after members of the community spread a message that the accelerometers record other personal data without the knowledge of the wearer. All of this made it difficult to conduct validation with accelerometers in this study population. However validation studies have been conducted with similar small samples of fewer than 30 (29,30). Because the study was limited to women, the findings are not generalizable to Indian men.

This is the first study to use accelerometry to validate GPAQ in India. Physical activity was assessed by using a culturally adapted GPAQ that we developed. Rigorous scoring protocols were used for both GPAQ and Actigraph data. This cultural adaptation of an instrument used worldwide to measure population-level physical activity will allow future studies in South India to use an appropriate instrument. The high correlation between overall minutes per week of MVPA obtained with the culturally adapted GPAQ and overall (nonbouted) accelerometer-based MVPA suggests that the modified GPAQ was sensitive to capturing the diverse nature of work done by women in South India. The Figure shows that in spite of varying over-estimation of self-reported physical activity compared with the accelerometer measurements, the modified GPAQ was sensitive in capturing data on both overall and bouted MVPA for all 24 participants in a consistent pattern.

In conclusion, this study points out the need to identify and incorporate local and sex- specific activities into self-reported physical activity measurement instruments for use in diverse settings, while being careful to maintain the key standardized properties of the instrument (eg, order of questions, wording) that allow for data comparability within and across countries. The adaptation of GPAQ through the processes reported in this study can be useful for improving the performance of the GPAQ in other settings with diverse cultures and their different types of work, travel, and leisure. Future studies are required to validate the original GPAQ with accelerometers to learn the extent of over-reporting and under-reporting of self-reported physical activity and to compare the findings with the modified GPAQ.

## Appendix: Modified Global Physical Activity Questionnaire

This appendix is available for download as a Microsoft Word document.
