# Supplementary material for: Adapting and Validating the Global Physical Activity Questionnaire (GPAQ) for Trivandrum, India, 2013
Source: Prev Chronic Dis. 2016 Apr 21;13:E53. doi: 10.5888/pcd13.150528 (PMC4854665; doi:10.5888/pcd13.150528)
Supplement: Supplementary file 1 [file 15_0528_Appendix.docx]

**MODIFIED GLOBAL PHYSICAL ACTIVITY QUESTIONNAIRE**

**(Instruction to the interviewer: Please ask the questions carefully and fill up the boxes appropriately)**

I am going to ask you about the time you spend doing different types of physical activity in a typical week. Please answer these questions even if you do not consider yourself to be a physically active person.

**At Work**

Think first about the time you spend doing work. Think of work as the things that you have to do such as paid or unpaid work, study/training, household chores, harvesting food/crops, fishing or hunting for food, seeking employment. *[Insert other examples if needed].* In answering the following questions 'vigorous-intensity activities' are activities that require hard physical effort and cause large increases in breathing or heart rate, 'moderate-intensity activities' are activities that require moderate physical effort and cause small increases in breathing or heart rate.

| Code | Questions | Response |  |
| --- | --- | --- | --- |
| P1 | Does your work involve vigorous-intensity activity that causes large increases in breathing or heart rate like *[carrying or lifting heavy loads, digging or construction work*] for at **least 10 minutes** continuously? | Yes 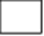  No 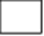 | If Yes Go to P1A  Else go to P2 |
| P1A | Please note the number of days a week and time spent each day on the following activities listed below which are considered to be vigorous. Write aside only those activities that is carried out by you in a **typical week** | | |

|  | **Activity** | **Number of days a week** | **Time spent in a day** |
| --- | --- | --- | --- |
| P1A1 | Carrying, loading or stacking wood | └─┴─┘ | hrs└─┴─┘:mins└─┴─┘ |
| P1A2 | Drawing water from the well and bringing water from other house | └─┴─┘ | hrs└─┴─┘:mins└─┴─┘ |
| P1A3 | Laying crushed rock | └─┴─┘ | hrs└─┴─┘:mins└─┴─┘ |
| P1A4 | Ural - Manual grinding | └─┴─┘ | hrs└─┴─┘:mins└─┴─┘ |
| P1A5 | Pounding grains | └─┴─┘ | hrs└─┴─┘:mins└─┴─┘ |
| P1A6 | Chopping wood-splitting logs | └─┴─┘ | hrs└─┴─┘:mins└─┴─┘ |
| P1A7 | Carrying heavy loads such as bricks | └─┴─┘ | hrs└─┴─┘:mins└─┴─┘ |
| P1A8 | Any other…………………………..  (Please specify) | └─┴─┘ | hrs└─┴─┘:mins└─┴─┘ |
| P1A9 | Any other…………………………..  (Please specify) | └─┴─┘ | hrs└─┴─┘:mins└─┴─┘ |
| P1A10 | Any other…………………………..  (Please specify) | └─┴─┘ | hrs└─┴─┘:mins└─┴─┘ |

| Code | Questions | Response |  |
| --- | --- | --- | --- |
| P2 | Does your work involve moderate-intensity activity that causes small increases in breathing or heart rate such as brisk walking *[or carrying light loads*] for at least 10 minutes continuously? | Yes 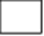  No 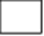 | If Yes Go to P2A  Else go to P3 |
| P2A | Please note the number of days a week and time spent each day on the following activities listed below which are considered to be moderate. Write aside only those activities that is carried out by you in a **typical week** | | |

| **P2A** | **Activity** | **Number of days a week** | **Time spent in a day** |
| --- | --- | --- | --- |
| P2A1 | Washing clothes | └─┴─┘ | hrs└─┴─┘:mins└─┴─┘ |
| P2A2 | Sweeping floor (inside or outside  house) | └─┴─┘ | hrs └─┴─┘: mins└─┴─┘ |
| P2A3 | Mopping floor( bend on knees and using hand) | └─┴─┘ | hrs └─┴─┘: mins └─┴─┘ |
| P2A4 | Drawing and bringing water from outside tap | └─┴─┘ | hrs └─┴─┘: mins └─┴─┘ |
| P2A5 | Animal care: feeding animals, washing  animals ,cleaning animal house, etc) | └─┴─┘ | hrs └─┴─┘: mins └─┴─┘ |
| P2A6 | Walking to bring grass, leaves, etc for feeding animals | └─┴─┘ | hrs └─┴─┘: mins└─┴─┘ |
| P2A7 | Milking cow | └─┴─┘ | hrs └─┴─┘: mins└─┴─┘ |
| P2A8 | Gardening: watering plants, pruning, sowing seeds, cleaning, etc | └─┴─┘ | hrs └─┴─┘: mins └─┴─┘ |
| P2A9 | Patient and elderly care | └─┴─┘ | hrs └─┴─┘: mins └─┴─┘ |
| P2A10 | Tailoring | └─┴─┘ | hrs └─┴─┘: mins └─┴─┘ |
| P2A11 | Child care-standing, dressing, bathing, grooming, feeding and occasional lifting of the child | └─┴─┘ | hrs └─┴─┘: mins └─┴─┘ |
| P2A12 | Multiple household task all at once-vigorous effort | └─┴─┘ | hrs └─┴─┘:mins └─┴─┘ |
| P2A13 | Sweeping the garage, sidewalk and outside the house | └─┴─┘ | hrs └─┴─┘:mins └─┴─┘ |
| P2A14 | Cooking or food preparation | └─┴─┘ | hrs └─┴─┘: mins └─┴─┘ |
| P2A15 | Shopping grocery without a grocery cart and carrying packages | └─┴─┘ | hrs └─┴─┘: mins └─┴─┘ |
| P2A16 | Any other……………….  (Please specify) | └─┴─┘ | hrs └─┴─┘:mins └─┴─┘ |
| P2A17 | Any other……………….  (Please specify) | └─┴─┘ | hrs └─┴─┘:mins └─┴─┘ |
| P2A18 | Any other……………….  (Please specify) | └─┴─┘ | hrs └─┴─┘:mins └─┴─┘ |

| Code | | | Questions | Response |  | | |
| --- | --- | --- | --- | --- | --- | --- | --- |
| P3 | | Do you walk or use a bicycle (*pedal cycle*) for at least 10 minutes continuously to get to and from places? | | Yes 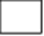  No 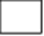 | If Yes go to P3A  If No, go to P 4 | | |
| **Walking to and from places**   \| **P3A** \| **Activity** \| **Number of days a week** \| **Time spent in a day** \| \| --- \| --- \| --- \| --- \| \| P3A1 \| To work \| └─┴─┘ \| hrs └─┴─┘: mins └─┴─┘ \| \| P3A2 \| To market \| └─┴─┘ \| hrs └─┴─┘: mins └─┴─┘ \| \| P3A3 \| To shops \| └─┴─┘ \| hrs └─┴─┘: mins └─┴─┘ \| \| P3A4 \| To bring children from school \| └─┴─┘ \| hrs └─┴─┘: mins └─┴─┘ \| \| P3A5 \| To see friends, relatives or others \| └─┴─┘ \| hrs └─┴─┘: mins └─┴─┘ \| \| P3A6 \| To temple or church \| └─┴─┘ \| hrs └─┴─┘: mins └─┴─┘ \| \| P3A7 \| Any other……………………  (Please specify) \| └─┴─┘ \| hrs └─┴─┘: mins └─┴─┘ \| \| **P3B Bicycling from and to places** \| \| \| \| \| P3B1 \| To work \| └─┴─┘ \| hrs └─┴─┘: mins └─┴─┘ \| \| P3B2 \| To market/shops \| └─┴─┘ \| hrs └─┴─┘: mins └─┴─┘ \| \| P3B3 \| Any other……………………  (Please specify) \| └─┴─┘ \| hrs └─┴─┘: mins └─┴─┘ \| \| P3B4 \| Any other……………………  (Please specify) \| └─┴─┘ \| hrs └─┴─┘: mins└─┴─┘ \| | | | | | | | |
| **Recreational activities**  The next questions exclude the work and transport activities that you have already mentioned.  Now I would like to ask you about sports, fitness and recreational activities (leisure) | | | | | | | |
| P4 | Do you do any vigorous-intensity sports, fitness or recreational (*leisure*) activities that cause large increases in breathing or heart rate like [*running or football, badminton,]* for at least 10 minutes continuously? | | | Yes 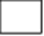  No 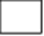 | | If Yes go to P5  If No, go to P7 | |
| P5 | In a typical week, on how many days do you do vigorous-intensity sports, fitness or recreational (*leisure*) activities? | | | Number of days└─┘ | | | |
| P6 | How much time do you spend doing vigorous-intensity sports, fitness or recreational activities on a typical day? | | | Hours : minutes └─┴─┘: └─┴─┘hrs: mins | | | |
| P7 | Do you do any moderate-intensity sports, fitness or recreational(leisure) activities that causes a small increase in breathing or heart rate such as brisk walking, cycling, swimming, volleyball for at least 10 minutes continuously? | | | Yes 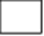  No 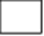 | | | If Yes go to P8  If No go to P10 |
| P8 | In a typical week, on how many days do you do moderate-intensity sports, fitness or recreational (leisure) activities | | | Number of days└─┘ | | | |
| P9 | How much time do you spend doing moderate-intensity sports, fitness or recreational (leisure) activities on a typical day? | | | Hours : minutes └─┴─┘: └─┴─┘ | | | |

| **Sedentary Behaviour:** The following question is about sitting or reclining at work, at home, getting to and from places, or with friends including time spent [sitting at a desk, sitting with friends, travelling in car, bus, train, reading, playing cards or watching television], but do not include time spent sleeping. | | |
| --- | --- | --- |
| P10 | How much time do you usually spend sitting or reclining on a typical day? | Hours : minutes└─┴─┘: └─┴─┘ |
| P11 | How many hours do you sleep on average during night? | Hours : minutes└─┴─┘: └─┴─┘ |
